# A basic need theory approach to problematic Internet use and the mediating effect of psychological distress

**DOI:** 10.3389/fpsyg.2014.01562

**Published:** 2015-01-14

**Authors:** Ting Yat Wong, Kenneth S. L. Yuen, Wang On Li

**Affiliations:** ^1^Department of Psychology, University of AmsterdamAmsterdam, Netherlands; ^2^Department of Counselling and Psychology, Hong Kong Shue Yan UniversityHong Kong, China; ^3^Focus Program Translational Neuroscience, Neuroimaging Center, Johannes Gutenberg University Medical CenterMainz, Germany

**Keywords:** self-determination theory, problematic Internet use, need satisfaction, psychological distress, structural equation modeling

## Abstract

The Internet provides an easily accessible way to meet certain needs. Over-reliance on it leads to problematic use, which studies show can be predicted by psychological distress. Self-determination theory proposes that we all have the basic need for autonomy, competency, and relatedness. This has been shown to explain the motivations behind problematic Internet use. This study hypothesizes that individuals who are psychologically disturbed because their basic needs are not being met are more vulnerable to becoming reliant on the Internet when they seek such needs satisfaction from online activities, and tests a model in which basic needs predict problematic Internet use, fully mediated by psychological distress. Problematic Internet use, psychological distress, and basic needs satisfaction were psychometrically measured in a sample of 229 Hong Kong University students and structural equation modeling was used to test the hypothesized model. All indices showed the model has a good fit. Further, statistical testing supported a mediation effect for psychological distress between needs satisfaction and problematic Internet use. The results extend our understanding of the development and prevention of problematic Internet use based on the framework of self-determination theory. Psychological distress could be used as an early predictor, while preventing and treating problematic Internet use should emphasize the fulfillment of unmet needs.

## INTRODUCTION

The development of the Internet has revolutionized the spread of information across the globe. Although the physical act of browsing the Internet could be largely solitary, the highly interactive nature of the Internet has created a virtual world-wide community. In this community people engage in a wide variety of online activities ranging from the exchange of information or ideas, socializing, and gaming, to more controversial activities like erotic interactions, gambling, and so on. The ease of accessibility of the Internet and the vast amount of activities have engaged a huge population, making the Internet a highly reinforcing and potentially addictive medium. The emergence of smartphones and tablet computers has further complicated the scenario. The round-the-clock availability of these mobile devices has blurred the boundary of Internet usage and it can be difficult for users to define when they are “logged in” to the Internet (Wallace, 2014). Mobile devices with Internet connectivity have become part of modern life (Hinić, 2011) and resulted in a large increase in online activities. Problematic Internet use has a potentially huge impact on individuals’ well-being (Young, 1998a). Problematic Internet use can create social disturbances as well as clinical issues, leading problematic users to experience academic, relational, financial, and occupational impairment as well as physical health problems in daily life. Despite all these consequences, whether or not problematic Internet use should be classified as a unidimensional psychiatric diagnosis is still being debated, given that Internet use is currently an umbrella term encapsulating a wide range of heterogeneous behaviors. So far, the only related disorder included by the American Psychiatric Association (APA) in the Diagnostic and Statistical Manual (DSM-V) is Internet gaming disorder, which has been identified as a potential pathological condition (that is, a condition requiring further study). Other pathological uses of the Internet fall into various diagnostic categories, such as sexual dysfunction and gambling disorder, based on the nature of the resulting behavior. Nonetheless, there seem to be generic factors underlying the diversity of problematic Internet behaviors. Laconi et al. (2014) review 45 assessment tools relating to problematic Internet use or addiction. Despite discrepancies in the definition and factor structures of these tools, two common factors emerged, namely negative outcomes and compulsive use (see also Wallace, 2014). These two factors align closely with recent neurological studies showing that excessive Internet use shares similar neurobiological mechanisms with substance and behavioral addiction (Yuan et al., 2011), which might in turn be related to dysfunctional impulse control (Shapira et al., 2000; Cao et al., 2007).

A more generic approach to understanding problematic Internet use across a wide range of online activities is to study the underlying motivations of users. Several researchers suggest that problematic Internet use stems from unmet real-life needs and that it is a way to relieve the problems encountered in daily life (Young, 1998a; Chak and Leung, 2004; Song et al., 2004). The Internet offers a highly accessible and immediate medium for users to satisfy such unmet needs (Wan and Chiou, 2006a). With reference to self-determination theory, needs can be grouped into three major types; relatedness, competence, and autonomy (Deci and Ryan, 2000; Ryan and Deci, 2000). Relatedness refers to a desirable attachment to others involving love and care; competence denotes satisfying a sense of mastery, and autonomy refers to gaining satisfaction from one’s own choices and decisions and their enactment in a way that is coherent with one’s integrated sense of self. All three types of needs have been shown to be instrumental in explaining the motivational components of individual behaviors in educational, healthcare, psychotherapeutic, and sporting settings. They are also instrumental in explaining Internet usage (Chen and Jang, 2010; Barnes and Pressey, 2011; Zhao et al., 2011; Wang, 2014). For instance, engagement in online social networks such as Facebook and Twitter can provide individuals with a sense of relatedness and autonomy by enabling them to connect with other people in a controlled manner (Wan and Chiou, 2006a; Sheldon et al., 2011; Nadkarni and Hofmann, 2012; Seidman, 2012). Participating in online gaming, on the other hand, could satisfy all three types of need via an intense interaction with other players (relatedness) to completing quests and achieving levels (competence), all through one’s own choices and decisions (autonomy; Kandell, 1998; Yee, 2006; Hsu et al., 2009). In general, the Internet has provided users with an immediate and easily accessible means to seek satisfaction and empowered them to control the way they present themselves regardless of their true identity or physical characteristics. It has thus become a very reinforcing stimulus.

The relationship between the satisfaction of needs and problematic Internet use can be further corroborated by the presence of psychological distress. In this context, psychological distress can be defined as general emotional disturbance related to negative mood, anxiety, and stress that most individuals will experience across their lifespan. Epidemiological studies show high levels of comorbidity between problematic Internet use and mood/anxiety disorders (Shaw and Black, 2006), and a positive correlation between psychological distress and severity of problematic Internet use (Young and Rogers, 1998; Caplan, 2002; Chak and Leung, 2004; Yuen and Lavin, 2004; Ebeling-Witte et al., 2007; Ceyhan and Ceyhan, 2008; Yeh et al., 2008). Davis’s (2001) cognitive behavioral model proposes that psychological distress, such as depression and anxiety, is an essential and significant catalyst of problematic Internet use. The psychological distress either develops from Internet use or exists long before this behavior is established, and can result from unmet needs (Ryan and Deci, 2000). For example, psychological distress can be associated with needs which are unmet as a result of shyness-induced social difficulties (Chak and Leung, 2004; Yuen and Lavin, 2004; Ebeling-Witte et al., 2007). Online communication provides shy individuals with a safety zone that enables them to avoid face-to-face interpersonal communication, freeing them from the negative and undesirable feelings associated with it. Individuals with poor oﬄine social relationships seek compensation from online interactions (Bessière et al., 2008). These individuals therefore may come to depend on the Internet to connect with others in order to satisfy their need for relatedness, potentially leading to excessive use (Chak and Leung, 2004; Ebeling-Witte et al., 2007).

**FIGURE 1:**
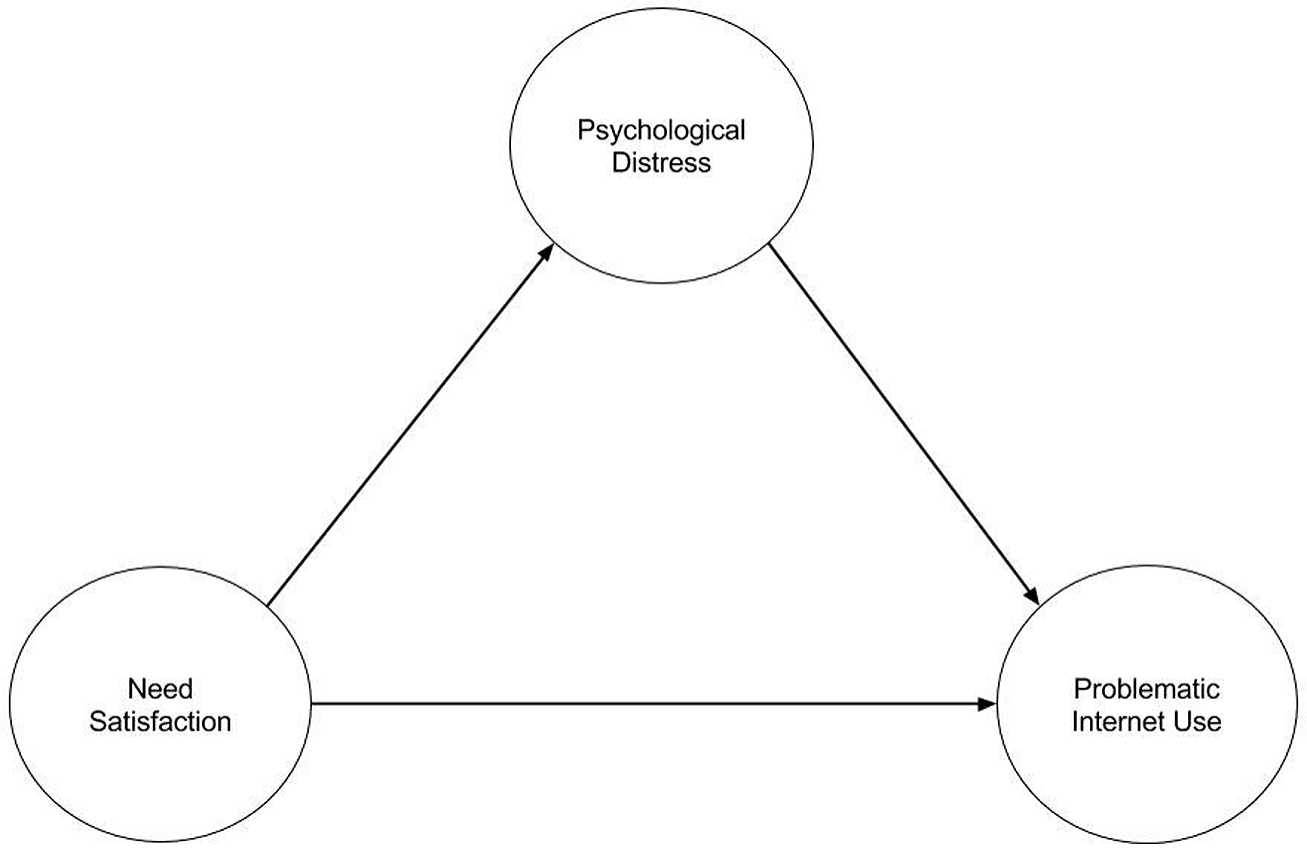
**The hypothesized model with the effect of need satisfaction to problematic Internet use mediated by psychological distress**.

This study examines generic motivation factors leading to problematic Internet use. Generic motivation factors are used since the Internet has become an everyday tool for satisfying a wide range of human needs, and self-determination theory has also been shown to be useful in explaining Internet behaviors. It remains unclear how well self-determination theory explains the motivational components of problematic Internet use, and whether psychological distress acts as an essential catalyst in its development. We hypothesize that psychological distress acts as a mediator in developing behavioral patterns of excessive Internet use. On this basis, we propose a theoretical model in which problematic Internet use is fully mediated by psychological distress and originates from unfulfilled basic psychological needs (see **Figure 1**) while the direct path from basic needs to problematic Internet use is expected to be non-significant. We recruited University students to test our model because they are among the heaviest users of the Internet and we expected a relatively high proportion of problematic users. In addition, the identity change which one undergoes in early adulthood poses a significant risk for psychological distress. Students are accordingly a suitable population on which to test our theoretical model (Morahan-Martin and Schumacher, 2000; Widyanto and Griffiths, 2006; Yen et al., 2009).

## MATERIALS AND METHODS

### PARTICIPANTS

A total of 250 questionnaires were distributed to undergraduates in a University in Hong Kong, of which 229 valid responses were returned. This was a cross-sectional design aiming at studying the problematic Internet use among young adults. The age of the participants ranged from 19 to 25 (*M* = 21.30, SD = 1.47). Of these, 72 were male, with a mean age of 21.35 (SD = 1.48) and 157 were female, with a mean age of 21.27 (SD = 1.47). The ethical standards of this study were approved by the Research Subcommittee of the Department of Counselling and Psychology, Hong Kong Shue Yan University. All participants were bilingual in English and Chinese, and they reported no problem in comprehending the questions.

### MEASURES

The questionnaire consisted of a battery of measures of problematic Internet use, needs satisfaction, and psychological distress. The original English versions were used to avoid inconsistent factor structures induced by the translation processes. Furthermore, the participants were all proficient in English and during the data collection process the experimenter received no requests for clarifications of the questionnaire items.

#### Problematic Internet use

This was measured using the Internet Addiction Test (Young, 1998b). Young (1998a) suggests that problematic Internet use shares characteristics with pathological gambling and firstly proposed the latter as the basis of assessment criteria. To enable a more accurate assessment, the Internet Addiction Test was then developed, including 20 items derived from the original diagnostic questionnaire (Young, 1998b). Factor analysis shows six factors related to excessive Internet use; salience, excessive use, neglect of work, anticipation, low control, and neglect of social life. Among all the available tools, the Internet Addiction Test is used most frequently (Laconi et al., 2014). It has been shown to have a stable factor structure in a range of studies (Chang and Law, 2008; Faraci et al., 2013) as well as satisfactory test–rest reliability (0.73–0.88) and good to excellent concurrent validity (0.46–0.90; Laconi et al., 2014). The Cronbach’s α ranges from 0.52 to 0.82 (Widyanto and McMurran, 2004), and for this study was 0.865 (subscales α = 0.48 to 0.74, except for the anticipation subscale, α = –0.06, probably due to this subscale consisting of only two items).

#### Psychological distress

Psychological distress was measured using the 21-item short version of the depression anxiety stress scales (DASS-21; Henry and Crawford, 2005). This comprises three 7-item subscales covering depression, anxiety, and stress. The DASS-21 has been shown to be reliable and has been validated in a non-clinical population. The Cronbach’s α of the subscales in this study were 0.806 (depression), 0.778 (anxiety), and 0.750 (stress).

#### Needs satisfaction

The general version of the basic psychological need satisfaction (BPNS) was used (Sheldon et al., 2001). It consists of a total of 21 items with three subscales; autonomy, competence, and relatedness. The Cronbach’s α values in this study were 0.467 (autonomy), 0.659 (competence), and 0.696 (relatedness).

### DATA ANALYSIS

A two-stage structural equation modeling (SEM) approach was used to evaluate the model (Anderson and Gerbing, 1988). A confirmatory factor analysis (CFA) was first conducted to examine whether or not the measurement model could provide an acceptable fit to the instruments used. This procedure confirmed the latent factor structure as intended to be measured by the assessment tools. The structural model for the hypothesized paths between the latent variables was then tested. For mediation analysis, the Sobel (1982) test and Tofighi and MacKinnon’s (2011) criteria were used to evaluate the effect. To further test the validity of the structural model, alternative models with different influence paths were tested and compared using the fit indices as well as the Akaike information criterion (AIC) and Bayesian information criterion (BIC). A combination of four indices was used to evaluate model fitness: chi-square, root mean square error of approximation (RMSEA), standardized root mean square residual (SRMSR), and comparative fit index (CFI). A non-significant chi-square value suggests the specified model is congruent with the observed data and is a reasonable measure of fit (Barrett, 2007). The RMSEA is considered to be adequate when it falls below 0.10. The SRMSR measures the standardized difference between the observed and predicted correlation. It is considered acceptable at values at 0.08 or less (Hu and Bentler, 1999). The CFI considers the number of parameters, or paths, in the model and is considered to be good at 0.90 or above. Using these indices as references, sample size estimation was performed using the method developed by Westland (2010). With a small anticipated effect size of 0.1, a conventional desired power of 0.8, the estimated smallest sample size required to detect an effect was *n* = 197. All SEM testing was performed with AMOS 20.0 and the other statistical analyses were conducted using SPSS.

## RESULTS

### DESCRIPTIVE STATISTICS

Mean, standard deviation, and correlation among the observed variables are shown in **Table 1**. Independent *t*-tests were carried out to examine any gender differences. There was no significant difference in terms of age or DASS-21 and BPNS scores (see **Table 2**), but male participants scored higher on the Internet Addiction Test (*t_227_* = 2.27, *p* = 0.02, *d* = 0.30) which is consistent with previous studies (see for example Chak and Leung, 2004; Chou et al., 2005).

**Table 1:** Mean, standard deviation, and correlation among the observed and latent variables.

Variable	*M*	SD	1	2	3	4	5	6	7	8	9	10	11	12
**Problematic Internet use**
(1) Salience	11.45	3.61	–	0.65**	0.53**	0.43**	0.63**	0.27*	0.52**	0.31**	0.32**	–0.22**	–0.28**	–0.22**
(2) Excessive use	13.19	3.41		–	0.51**	0.41**	0.62**	0.25**	0.28**	0.18**	0.19**	–0.14**	–0.13**	–0.06
(3) Neglect of work	7.22	2.64			–	0.41**	0.74**	0.15**	0.43**	0.25**	0.28**	–0.21**	–0.25**	–0.12
(4) Anticipation	5.94	1.59				–	0.42**	0.10	0.20**	0.13**	0.20**	–0.13*	–0.16*	0.11
(5) Lack of control	7.48	2.46					–	0.23**	0.38**	0.19**	0.29**	–0.13	–0.20**	0.003
(6) Neglect of social life	4.19	1.64						–	0.12	0.14*	0.01	–0.04	0.04	–0.14*
**Psychological distress**
(7) Depression	12.80	7.67							–	0.68**	0.64**	–0.48**	–0.41**	–0.38**
(8) Anxiety	13.79	7.92								–	0.64**	–0.42**	–0.37**	–0.27**
(9) Stress	17.40	7.97									–	–0.46**	–0.34**	–0.13*
**Need satisfaction**
(10) Autonomy	30.26	3.97										–	0.59**	0.40**
(11) Competence	26.02	4.50											–	0.38**
(12) Relatedness	39.79	5.30												–

*Correlations are significant at a 0.05 level, *p* < 0.05; **correlations are significant at a 0.01 level, *p* < 0.01.

**Table 2:** Descriptive statistics and pair-wise comparisons between the two gender groups.

Variables	Male	Female			
*n*	72	157			

	***Mean***	***Mean***	***t_**227**_***	***p***	***Cohen’s d***

Internet Addiction Test	52.50	48.81	2.27	0.02*	0.32
Time spent (h/per week)	30.49	22.86	3.63	<0.01**	0.47
Autonomy	30.81	30.01	1.41	0.16	0.20
Competence	26.21	25.94	0.42	0.67	0.06
Relatedness	39.03	40.13	1.47	0.14	0.21
Depression	14.00	12.25	1.60	0.11	0.22
Anxiety	13.00	14.15	1.02	0.31	0.15
Stress	16.19	17.95	1.55	0.12	0.22

*Significant at a 0.05 level, *p* < 0.05; **significant at a 0.01 level, *p* < 0.01.

Participants in this study had moderate Internet Addiction Test scores (*M* = 49.97) and long Internet use times (*M* = 25.26 h per week), as compared with data from previous population surveys or normative data. The Internet Addiction Test mean scores of both male and female respondents fell within the frequent (40–69) range of problematic Internet use (Young, 1998a; Laconi et al., 2014). Although this range categorization is arbitrary, the average hours of Internet use per week of these participants (males 30.49 h; females 22.86 h) fell into the at-risk range as determined by the criteria developed in one population survey in Shanghai (Xu et al., 2012). With reference to data from a recent population study of 12,446 high school students in nearby Guangdong Province, who shared a common language and cultural characteristics with the sample in this study, these participants’ amount of Internet use placed them above the 67th percentile in terms of normative Internet usage (Wang et al., 2011). These findings provide converging evidence of frequent Internet usage among our current sample. Although there is yet a consensual cutoff score defining problematic Internet use, the descriptive statistics imply a large part of the study population exhibits at least some degree of problematic use.

### MODEL TESTING

**FIGURE 2:**
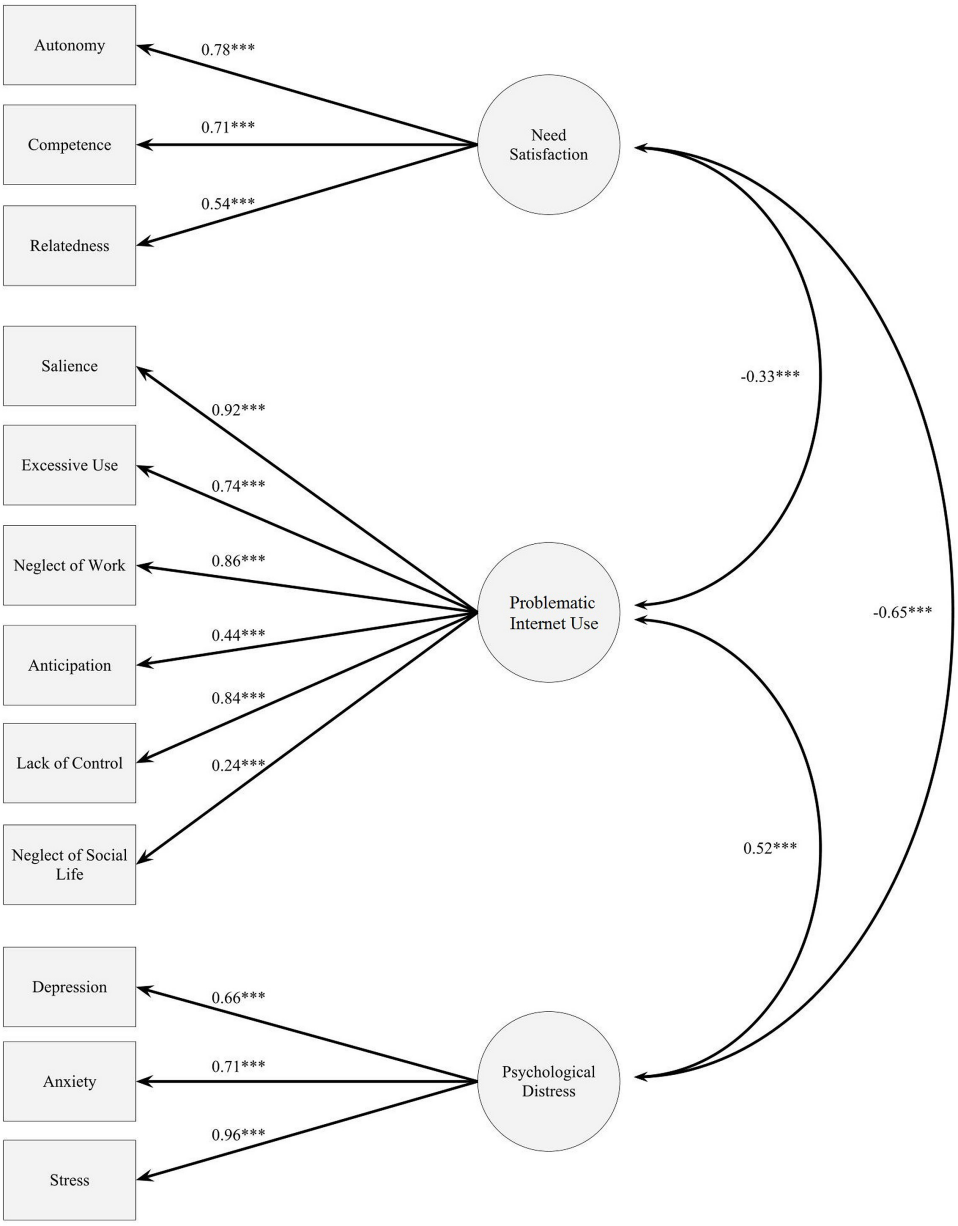
**The measurement model among need satisfaction, problematic Internet use and psychological distress.** ***Path coefficients are significant at a 0.001 level, *p* < 0.001.

The chi-square of the measurement model was 104.03 (*df* = 45, *n* = 229), with a *p* < 0.01. This initial result demonstrated that it was a moderately good fit, with RMSEA = 0.09 < 0.10, SRMR = 0.06 < 0.08, and CFI = 0.95 > 0.90. All the factor loadings of the different latent variables were significant. A detailed measurement model is shown in **Figure 2**. This result suggested that the model fit was acceptable and further evaluation would be worthwhile.

**FIGURE 3:**
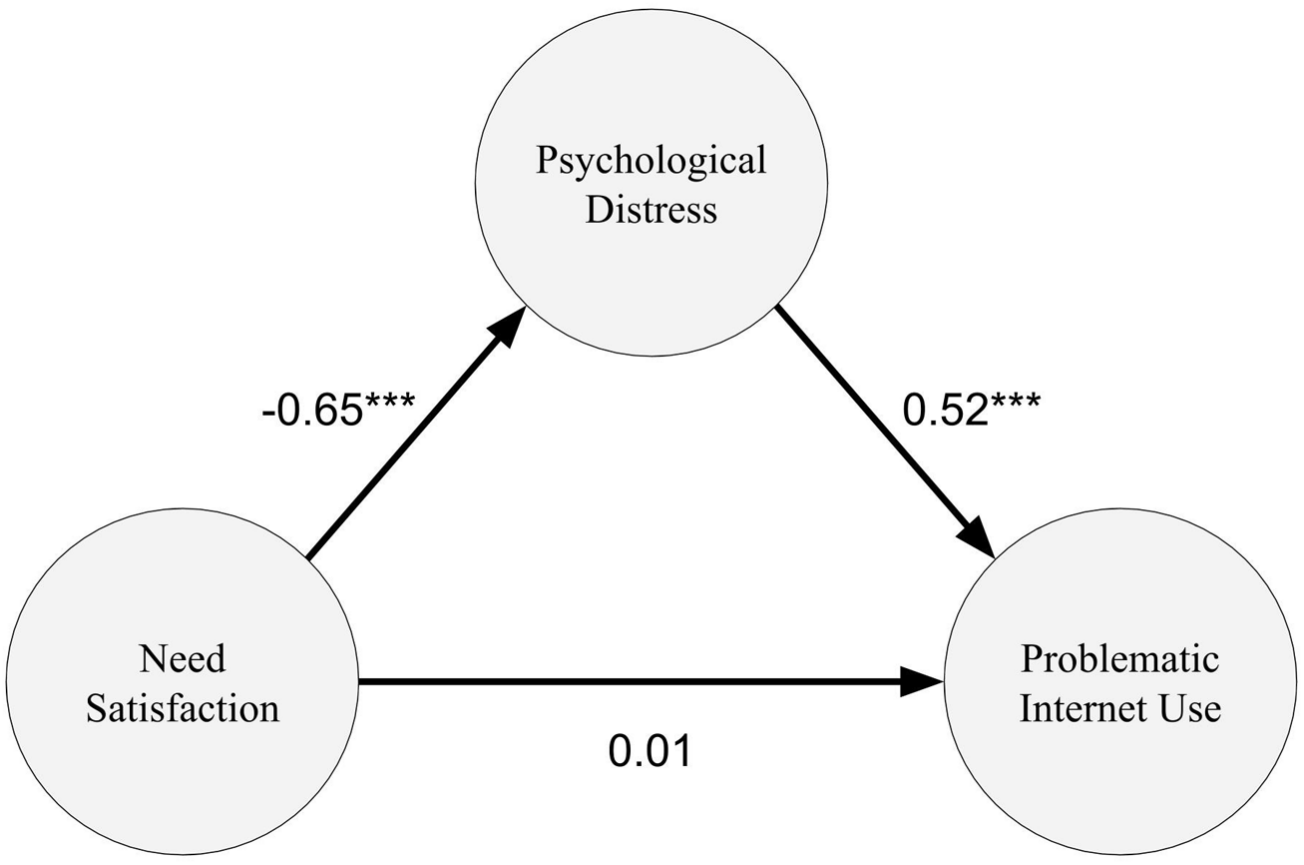
**The structural model of the influence of need satisfaction to problematic Internet use with mediating effect of psychological distress.** ***Path coefficients are significant at a 0.001 level, *p* < 0.001.

The maximum likelihood method was used to explore the fitness of the structural model (see **Figure 3**). The result showed that the model has a relatively good fit, with chi-square (45, *n* = 229) = 104.03 at *p* < 0.001, RMSEA = 0.08 < 0.10, SRMR = 0.06 < 0.08, CFI = 0.95 > 0.90, AIC = 170.03, and BIC = 283.35. The model accounted for 27% of the variance in problematic Internet use. A power calculation using the method developed by MacCallum et al. (1996) revealed satisfactory power at π = 0.71. As predicted, the only significant path to problematic Internet use from needs satisfaction was mediated by psychological distress. All the structural paths were significant at the 0.001 level, except for the path leading from the satisfaction of basic needs to problematic Internet use.

To statistically examine the proposed mediating effect, the Sobel (1982) test was used. The result supported the mediating effect of psychological distress between needs satisfaction and problematic Internet use (*Z* = 2.41, *p* = 0.02 < 0.05). A further test using Tofighi and MacKinnon’s (2011) criteria confirmed the existence of a significant mediating effect (mean mediation term = –0.44, 95% C.I. = –0.24 to –0.68).

### ALTERNATIVE MODELS

Three alternatives were tested to further confirm our theoretical model (**Table 3**). The first was a model in which psychological needs satisfaction and psychological distress both contributed to a direct path to problematic Internet use, with the mediating path from needs satisfaction to psychological distress omitted. Compared to the hypothesized theoretical model with the mediating path, this model has a poorer fit (model A1). All the fit indices and AIC/BIC suggested that the hypothesized model had a better fit, providing support for the theoretical model.

**Table 3:** Summary of model fit indices and mediation tests of the theoretical model and the three alternative models (A1: without a mediating path from need satisfaction to psychological distress; A2: removed subscales with low reliability, autonomy in BPNS and anticipation in Internet Addiction Test; A3: treating both BPNS and Internet Addiction Test as unitary constructs).

	Theoretical model	A1	A2	A3
χ*^2^ (p-value)*	104.032 (<0.001)	178.315 (<0.001)	60.233 (<0.001)	3.421 (0.331)
*df* of χ*^2^*	45	46	26	3
RMSEA (<0.08)	0.078	0.112	0.076	0.025
SRMR (<0.08)	0.0586	0.145	0.0505	0.0187
CFI (>0.9)	0.984	0.886	0.964	0.999
AIC	170.032	242.315	118.233	27.421
BIC	283.345	352.194	217.811	68.626
Sobel test Z *(p-value)*	2.412 (0.016)	n.a.	–2.49 (0.013)	–2.603 (<0.001)
Mean mediation term	–0.44	n.a.	–0.224	–0.267
95% C.I.	–0.241 to –0.678	n.a.	–0.063 to –0.419	–0.069 to –0.473

*Unless otherwise specified, numbers in brackets state the criteria used in article*.

Despite the theoretical model confirming the validity of the latent variables, low levels of internal consistency were observed in the anticipation subscale of the Internet Addiction Test and the autonomy subscale of the BPNS. Both subscales were used in this analysis because studies have demonstrated their utility across different countries. For instance, autonomy is suggested to be a universal need (Ryan and Deci, 2003; Vansteenkiste et al., 2006; Rudy et al., 2007; Chirkov, 2009). While the anticipation subscale only consists of two items and, hence, is intrinsically prone to generating unstable statistics, the autonomy subscale could be attributed to a different cultural perception of the constructs among Hong Kong adolescents (Iyengar and Lepper, 1999; Chang and Law, 2008; Markus and Schwartz, 2010). For instance, it has been reported that Hong Kong young people expect to gain autonomy at a significantly later age than their western counterparts (Feldman and Rosenthal, 1991). Compared to the western population, the developmental delay in terms of Hong Kong students’ expectation for autonomy may correspondingly influence their needs. The higher variance in their need for autonomy may thus lead to a lower internal consistency for this subscale. To rule out the potential confounding effect due to the low internal consistency of these two subscales, two additional alternative models were tested (**Table 3**); one with these two subscales removed altogether (model A2) and the other treating both BPNS and Internet Addiction Test as measures of unitary latent constructs (model A3). If the low internal consistency of these two subscales was genuinely a threat to the validity of the original hypothesized model, removing them (model A2) should significantly change the path coefficient. On the other hand, collapsing all the subscales into one latent variable (model A3) should enhance internal consistency. If the alternative model A3 revealed significantly different path coefficients, this would indicate that the effect of the autonomy and anticipation subscales in the original model could not be relied upon. The results showed that both alternatives demonstrated comparable fitness when compared to the hypothesized structural model, and the mediation effect of psychological distress remained significant (**Table 3**). This shows that the internal consistency does not pose a threat to the hypothesized model.

### DEGREE OF PROBLEMATIC INTERNET USE

As mentioned above, the Internet Addiction Test scores of these participants were in the moderate range. It is also of interest to explore whether the hypothesized model holds for users demonstrating different levels of problematic Internet use. Thus, the sample was median split into two groups (light users vs. heavy users) according to their Internet Addiction Test scores (*M* = 49.97). **Table 4** summarizes their differences in terms of the variables of interest. Participants in the heavy user group consistently spent more time using the Internet compared to the light user group (*t_227_* = 4.94, *p* < 0.01, *d* = 0.65). They also suffered from poorer psychological health (depression *t_227_* = –5.16, *p* < 0.01, *d* = 0.68; anxiety *t_227_* = 3.19, *p* < 0.01, *d* = 0.35; and stress *t_227_* = 3.67, *p* < 0.01; *d* = 0.48) and scored significantly lower in satisfaction of two of the basic needs, namely autonomy (*t_227_* = 2.54, *p* < 0.01, *d* = 0.34) and competence (*t_227_* = 2.63, *p* = 0.01, *d* = 0.35).

**Table 4:** Descriptive statistics and pair-wise comparisons between the two problematic Internet use groups.

Variables	Light	Heavy			
*n*	117	112			

	***Mean***	***Mean***	***t_**227**_***	***p***	***Cohen’s d***

Internet Addiction Test	40.74	59.62	21.73	<0.01**	2.87
Time spent (h/per week)	20.63	30.09	4.94	<0.01**	1.93
Autonomy	30.91	29.59	2.54	0.01*	0.34
Competence	26.78	25.23	2.63	<0.01**	0.35
Relatedness	40.09	39.46	0.90	0.37	0.12
Depression	10.38	15.34	5.16	<0.01**	0.68
Anxiety	12.19	15.46	3.19	<0.01**	0.42
Stress	15.56	19.32	3.67	<0.01**	0.48

*Significant at a 0.05 level, *p* < 0.05; **significant at a 0.01 level, *p* < 0.01.

The possibility of whether the hypothesized model fits both groups equally well was explored by testing a multigroup nested model. **Table 5** summarizes the overall model fitness and path coefficients between the three latent variables for both groups. Consistent with the hypothesized model, the indirect path between basic needs and problematic Internet use was significantly mediated by psychological distress in the heavy users group only. In contrast, the path coefficients pattern was different for the light users group. Psychological distress still significantly predicted problematic Internet use and the direct path between basic needs and problematic Internet use was marginally significant (*p* = 0.050). There was no significant mediation effect.

**Table 5:** Goodness-of-fit indices and path coefficients of the multi-group nested model.

χ^2^ *(p-value)*	*161.008 (p < 0.001)*		
*df* of χ*^2^*	*90*		
RMSEA (<0.08)	0.073		
SRMR (<0.08)	0.0995		
CFI (>0.9)	0.84		
AIC	293.008		
BIC	310.094		

	**Light users**	**Heavy users**

**Path coefficients**
Needs satisfaction > psychological distress	–0.601***	–0.748***
Psychological distress > problematic Internet use	–0.072	0.617***
Needs satisfaction > problematic Internet use	–0.136	0.060

***Denotes significant path at *p* < 0.001.

## DISCUSSION

This study addressed data from University students who used the Internet frequently and likely suffered some degree of problematic Internet use. SEM was applied in order to examine the interplay between psychological needs satisfaction, psychological distress, and problematic Internet use the results support the proposition that self-determination theory is instrumental in explaining the motivational components of problematic Internet use, and their relationship is mediated by psychological distress. Needs satisfaction was significantly and negatively associated with psychological distress, which itself was significantly and positively associated with problematic Internet use. This conforms to the hypothesized mediating role of psychological distress in problematic Internet use (Young, 1998b; Davis, 2001; Yeh et al., 2008; Wang et al., 2011).

Studies show that individuals will attempt to satisfy their needs through participating in different kinds of Internet activities (Kandell, 1998; Wan and Chiou, 2006a,b; Yee, 2006; Hsu et al., 2009; Back et al., 2010; Huang, 2010; Barnes and Pressey, 2011; Sheldon et al., 2011; Nadkarni and Hofmann, 2012; Seidman, 2012). The emergence of mobile technologies has broadened the functionality and utility of the Internet, thereby providing people with an easily accessible and immediate means to satisfy their basic needs. In this context, these findings extend our understanding of what motivates people to get involved in Internet activities in general. Based on the framework of self-determination theory, individuals look for ways to satisfy their basic needs, autonomy, competency, and relatedness online. If an individual fails to fulfill his or her basic needs, the level of psychological distress increases (Ryan and Deci, 2000). Such individuals are more likely to turn to Internet activities for compensation (Song et al., 2014) and may develop problematic use patterns. This is consistent with the comorbidity observed between mood/anxiety disorders and problematic Internet use in previous work (Shaw and Black, 2006) and with a recent report by Dong et al. (2011) suggesting symptoms relating to psychological distress significantly predict problematic Internet usage.

Using the Internet, especially via mobile devices, can be an immediate way to satisfy basic needs. Individuals feeling distress are more likely to make use of this approach (Song et al., 2014). Such individuals are not satisfied with conventional means and, as a result, Internet activities provide them an alternative way to gain satisfaction (Yuen and Lavin, 2004). Some users, however, being poorly adapted to this alternative method, fail to derive satisfaction from Internet activities thus leaving their needs unresolved. For example some Internet users receiving high levels of online social support do not feel their emotional loneliness alleviated (Hardie and Tee, 2007). In such circumstances, individuals’ unmet needs are unresolved and they may suffer from increased psychological distress which in turn motivates them to go online more often to satisfy their needs, creating a vicious cycle of Internet reliance. The development of reliance implies that the Internet is more than just a tool or a leisure activity, with users starting to develop compulsions as well as neglecting real-life issues. A number of studies show a significant association between problematic Internet use and escapism (Yee, 2006; Kuss et al., 2012). Individuals may use the Internet to escape the cognitive and emotional challenges arising from unsatisfying life circumstances oﬄine (Henning and Vorderer, 2001). This hinders them from finding other ways to resolve their unmet needs and blocks effective intervention, further promoting the development of problematic Internet use (Ko et al., 2012).

The exploratory analysis and split-half model fitting presented here provide additional evidence for the proposed mechanism. Pairwise comparison indicated significant differences in basic needs satisfaction and psychological distress between the light and heavy user groups. The former demonstrated a marginally significant association between needs satisfaction and problematic Internet use. This supports the proposition that Internet users are motivated by an urge to fulfill their needs when they first engage in online activities. By contrast, the heavy user group showed a significant mediation effect for psychological distress, similar to that shown in the main analysis. Despite the reduced statistical power in the split-half analysis, the model was still a reasonable fit. Together with the observed differences in terms of psychological distress across the two groups, this exploratory analysis suggests that the amount of Internet use is associated with a reduction in basic needs satisfaction when usage is within the normal range (light users group). When Internet usage reaches a problematic level, this association is mediated by an increase in psychological distress.

An alternative explanation for our findings is that problematic Internet use significantly worsens psychological needs satisfaction. With the correlational nature of SEM, we could not rule out the possibility that problematic Internet use reciprocally influences psychological needs satisfaction. In fact, this reciprocal influence is a potential basis for the development of a vicious cycle. This alternative explanation should be further tested by studies adopting experimental manipulation on the amount of Internet use and examining psychological needs satisfaction as the outcome measure.

Psychological distress helps to identify individuals at risk of developing problematic Internet usage. Nevertheless, the results reported here suggest that measures for relieving psychological distress (Caplan, 2002; The Center for Internet Addiction, 2009) may not be enough to prevent and treat Internet addiction. An additional and alternative measure to help clients escape from the grip of the Internet might actually be to help them meet their real-life basic needs. One specific example is facilitating the improvement of social skills by individuals with social difficulties. This may ease problematic Internet use among such persons by fulfilling their needs of competence and relatedness through participation in social groups (Yeh et al., 2008). This will, in turn, allow these individuals to gain satisfaction from real-life situations.

### LIMITATIONS

Problematic Internet use is examined as a generic and integrated behavior in the present study, since the main aim was to test the basic motivational underpinnings. Nevertheless, there are specific types of addictive online behaviors which have been shown to be associated with specific cognitive deficits, such as impulse control difficulties (The Center for Internet Addiction, 2009). These findings do not exclude the role of such cognitive deficits in the development of specific addictive online behaviors. Future research could examine whether basic needs satisfaction and psychological distress could be incorporated in a model of these cognitive deficits to better predict the course of specific online addictive behaviors.

The assessment tools for problematic Internet use have been widely criticized due to the dynamic change of Internet environment. Though the Internet Addiction Test is the most widely used, its factor structure varies across studies. A similar issue with the internal consistency of subscales has been observed in this sample, although the potential confounding effect has been excluded by testing alternative models. In addition, some of the Internet Addiction Test items are now outdated (e.g., evaluating e-mail use but not instant messages; Laconi et al., 2014). Future studies should look at developing an updated assessment tool to better evaluate Internet use so as to facilitate research on the psychopathology of problematic use. A properly validated assessment tool would also help to identify clinical cases.

The sample used in this study, due to age and developmental stage, represents only a cross-sectional cohort of the population that presents with high risk of problematic Internet use. Most participants in this study were frequent users. The exploration of the split-half analysis was restricted by the reduced statistical power and the arbitrary segregation between light and heavy users. Accordingly, these findings are not generalizable to the general etiology of problematic Internet use and the use of SEM with cross-sectional data forbids examination of causality. A stratified, population-based study should be conducted to establish a generic etiology model for problematic Internet use. In addition, a longitudinal study would provide further empirical evidence about the causal relationship of the tested constructs.

## CONCLUSION

Internet activities can provide a sense of satisfaction of basic psychological needs including competency, relatedness, and autonomy. Individuals who have developed psychological distress due to these unmet needs are particularly vulnerable to problematic Internet use. The compulsion to use the Internet also blocks individuals from looking for other way to resolve their distress, which in turn accelerates and intensifies the degree of problematic use. This study has extended self-determination theory to explain the motivational components of problematic Internet use, and provided empirical support for the mediating role of psychological distress between needs satisfaction and problematic Internet use. Effective interventions should therefore address the individual’s motivational needs as well as his or her psychological distress.

## Conflict of Interest Statement

The Associate Editor Reinout W. Wiers declares that, despite being affiliated to the same institution as author Ting Yat Wong, the review process was handled objectively and no conflict of interest exists. The authors declare that the research was conducted in the absence of any commercial or financial relationships that could be construed as a potential conflict of interest.
